# Unravelling nicotinic receptor and ligand features underlying neonicotinoid knockdown actions on the malaria vector mosquito *Anopheles gambiae*

**DOI:** 10.1098/rsob.240057

**Published:** 2024-07-24

**Authors:** Ryo Ito, Masaki Kamiya, Koichi Takayama, Sumito Mori, Rei Matsumoto, Mayuka Takebayashi, Hisanori Ojima, Shota Fujimura, Haruki Yamamoto, Masayuki Ohno, Makoto Ihara, Toshihide Okajima, Atsuko Yamashita, Fraser Colman, Gareth J. Lycett, David B. Sattelle, Kazuhiko Matsuda

**Affiliations:** ^1^ Department of Applied Biological Chemistry, Faculty of Agriculture, Kindai University, 3327-204 Nakamachi, Nara 631-8505, Japan; ^2^ Agricultural Technology and Innovation Research Institute, Kindai University, 3327-204 Nakamachi, Nara 631-8505, Japan; ^3^ Institute of Scientific and Industrial Research, Osaka University, 8-1 Mihogaoka, Ibaraki, Osaka 567-0047, Japan; ^4^ Graduate School of Medicine, Dentistry and Pharmaceutical Sciences, Okayama University, 1-1-1 Tsushima-naka, Kita-ku, Okayama 700-8530, Japan; ^5^ Liverpool School of Tropical Medicine, Pembroke Place, Liverpool L3 5QA, UK; ^6^ Centre for Respiratory Biology, UCL Respiratory, Division of Medicine, University College London, London WC1E 6JF, UK

**Keywords:** *Anopheles gambiae*, knockdown, malaria, neonicotinoids, nicotinic acetylcholine receptors

## Abstract

With the spread of resistance to long-established insecticides targeting *Anopheles* malaria vectors, understanding the actions of compounds newly identified for vector control is essential. With new commercial vector-control products containing neonicotinoids under development, we investigate the actions of 6 neonicotinoids (imidacloprid, thiacloprid, clothianidin, dinotefuran, nitenpyram and acetamiprid) on 13 *Anopheles gambiae* nicotinic acetylcholine receptor (nAChR) subtypes produced by expression of combinations of the Ag*α*1, Ag*α*2, Ag*α*3, Ag*α*8 and Ag*β*1 subunits in *Xenopus laevis* oocytes, the *Drosophila melanogaster* orthologues of which we have previously shown to be important in neonicotinoid actions. The presence of the Ag*α*2 subunit reduces neonicotinoid affinity for the mosquito nAChRs, whereas the Ag*α*3 subunit increases it. Crystal structures of the acetylcholine binding protein (AChBP), an established surrogate for the ligand-binding domain, with dinotefuran bound, shows a unique target site interaction through hydrogen bond formation and CH-N interaction at the tetrahydrofuran ring. This is of interest as dinotefuran is also under trial as the toxic element in baited traps. Multiple regression analyses show a correlation between the efficacy of neonicotinoids for the Ag*α*1/Ag*α*2/Ag*α*8/Ag*β*1 nAChR, their hydrophobicity and their rate of knockdown of adult female *An. gambiae*, providing new insights into neonicotinoid features important for malaria vector control.

## Graphical abstract

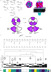

## 1. Introduction

Malaria is endemic to many regions of sub-Saharan Africa, as well as several parts of Southeast Asia and South America. Currently, chemotherapy, immunization by the RTS, S vaccine against the *Plasmodium falciparum* parasite, the deployment of insecticide-treated nets (ITNs) and indoor residual spraying (IRS) targeting mosquito vectors of the genus *Anopheles* are important strategies for disease control [1]. The insecticide-based measures alone averted 600 million malaria cases between 2000 and 2015 [2], but this trend of falling cases has plateaued and reversed recently, partly due to the increased insecticide resistance (IR) [1] of mosquito vectors. By 2022, global malaria cases rose to greater than 200 million [1], leading to more than 600 000 deaths.

To date, *Anopheles* insecticidal control has relied heavily on pyrethroids[3] which modulate insect sodium channels, and IR has developed through target site mutations [4–7], enhanced metabolism [8–10], thickening and chemical component changes of vector cuticle [11], as well as increased expression of the sensory appendage protein (SAP2) with its potential capacity to bind pyrethroids [12]. One approach being taken by control programmes is exploiting proven insecticides with alternative modes of action that have not previously been used in public health. Among such insecticidal candidates, clothianidin, a neonicotinoid insecticide, is being explored for IRS use and is found to be effective on pyrethroid-resistant strains of *Anopheles* mosquitoes [13].

**Figure 1.**
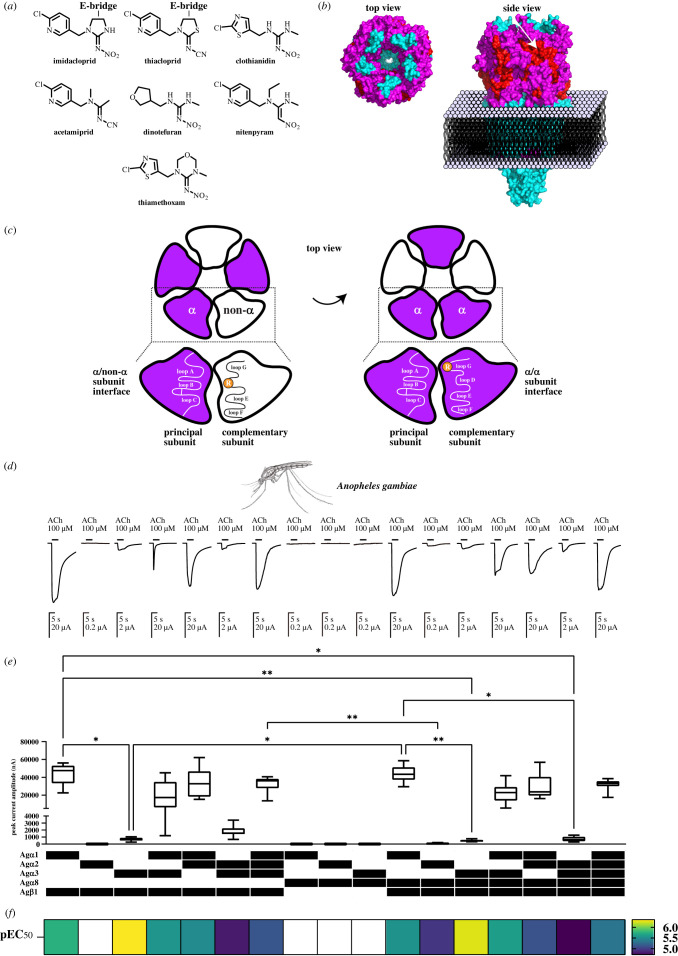
Neonicotinoids, their targets (nAChRs) and the functional expression of *An. gambiae* nAChRs in *X. laevis* oocytes, with the aid of cofactors AgRIC-3, AgUNC-50 and AgTMX3, measured by their responses to the neurotransmitter ACh. (*a*) Structure of neonicotinoids in IRAC group 4A. Imidacloprid and thiacloprid possess an ethylene bridge (E-bridge), while others have no E-bridge. (*b*) Top left edge and side-views of a nAChR structure where helices, loop and sheets are coloured cyan, magenta and red, respectively. The figure was illustrated by PyMol software (Schrödinger, USA) using the protein data base file 2BG9. The orthosteric site (ACh and neonicotinoid binding domain) is arrowed. (*c*) Schematic representations of the orthosteric sites formed at *α*/non-α and *α*/*α* subunit interfaces. Loops A, B, C, D, E, F and G involved in the interactions with ACh and neonicotinoids are shown. Basic residues (arginines) in loops D and G underpinning electrostatic interactions with the nitro or cyano groups (see panel A for the functional groups) are highlighted. (*d*) Responses to 100 µM ACh recorded from *X. laevis* oocytes injected with the subunit cRNAs together with the cofactor cRNAs. (*e*) Current amplitude of the responses 100 µM ACh of *X. laevis* oocytes injected with the subunit and cofactor cRNAs. Each box plot represents the 75 and 25% percentiles of data and horizonal line in each box indicates the median of data (*n* = 10 oocytes, from two frogs). Asterisks * and ** indicate that the differences are significant at levels of *p* < 0.05 and < 0.01, respectively (one-way ANOVA, Kruskal–Wallis test). The Ag*β*1 subunit is essential for the functional expression and the Ag*α*1 subunit enhanced the amplitude of the ACh-induced response. (*f*) Heatmap representation of pEC_50_ values of ACh for the 13 *An. gambiae* nAChRs. White area means that the value could not be determined because the nAChR was not robustly expressed in the oocytes. The expressed nAChRs display diverse ACh sensitivity.

Neonicotinoids are a major insecticide class, displaying high selectivity to insects over vertebrates and have been widely used in pest control (figure 1*a*) [14–19]. They are modulators of insect nAChRs [14–19], exhibiting partial, full and super agonist actions on native insect [20,21] and recombinant insect nAChRs [22,23] and act as antagonists at low concentration [22,24], underpinning sublethal effects. Neonicotinoids bind to the orthosteric sites (normally occupied by the neurotransmitter ACh) of nAChRs at *α*/non-α or *α*/*α* subunit interfaces formed by seven loops (A, B, C, D, E, F and G) in the long N-terminal, extracellular domain (figure 1*b,c*) [15,25–27]. Several amino acids involved in nAChR-neonicotinoid interactions have been identified [28–33]. Of particular importance are the basic residues in loops D and G, which contribute to the selective actions of neonicotinoids on insect nAChRs [28,30,33].

With increased neonicotinoid use, potential adverse effects on non-target organisms such as pollinators have been described [15,34] and in areas where neonicotinoids are also widely used in agriculture, a low level of resistance has been reported in *Anopheles* [35,36]. The use of certain neonicotinoids is now restricted for agricultural pest control in the EU [15,34]. Nevertheless, the targeted deployment of clothianidin for IRS based vector control is under investigation, because using neonicotinoids indoors could severely reduce exposure to pollinators, a persistent problem with neonicotinoids. For example, Fludora Fusion (a deltamethrin/clothianidin combined treatment from Bayer) and Sumishield 50WG (a clothianidin formulation from Sumitomo Chemical) are prequalified by the WHO pesticide evaluation scheme [37]. Moreover, dinotefuran is being trialed as the lethal component of attractive toxic sugar bait strategies targeting outdoor biting mosquitoes [38]. Hence, elucidating the molecular mechanism of action of clothianidin, dinotefuran and other neonicotinoids on their molecular targets in the *Anopheles* malaria vectors is urgently needed to predict and allow monitoring for target site resistance that could emerge.

Until recently, it had been challenging to express functional insect nAChRs robustly in cell lines and *Xenopus laevis* oocytes, thereby limiting our understanding of neonicotinoid actions on insect nAChRs. The discovery that a thioredoxin-related transmembrane protein (TMX3) was key to enabling robust functional expression of insect nAChRs in *X. laevis* oocytes [16,39,40] led to the characterization of the agonist and antagonist actions of imidacloprid, thiacloprid and clothianidin on fruit fly *Drosophila melanogaster*, honeybee *Apis mellifera* and bumblebee *Bombus terrestris* nAChRs [39]. Fascinatingly, all three neonicotinoids not only activated the pollinator nAChRs at nanomolar concentrations or higher, directly inducing death, but significantly suppressed the receptor response to ACh at picomolar concentrations (much lower than field-relevant concentrations) [39]. Such sublethal effects of some neonicotinoids can influence the microglomerular density of mushroom bodies [41] and Kenyon cells [42], as well as nAChR response amplitude and subunit combination [43], memory, circadian behaviour, sleep and foraging rhythms [44–46], counselling caution in their continued use for crop protection.

In *D. melanogaster*, D*α*1, D*α*2, D*α*3, D*β*1 and D*β*2 subunits coexist in certain neurons and comprise neonicotinoid-sensitive nAChR subtypes [39,47], while D*α*5−D*α*7 subunits form different nAChR subtypes. For example, the D*α*5/D*α*6 nAChR is not responsive to imidacloprid but is sensitive to spinosad [48]. We found that the toxicity of neonicotinoids is the result of complex actions on diverse nAChR subtypes. Lowering the expression of the D*α*2 nAChR subunit resulted in hyper-sensitivity to neonicotinoids in adult males and females of *D. melanogaster* [47]. However, there is no information on how subunits and neonicotinoid structures affect target site actions and toxicity in the *Anopheles* malaria vectors.

Hence, we investigated the agonist actions of the IRAC class 4A commercial neonicotinoids (imidacloprid, thiacloprid, clothianidin, acetamiprid, dinotefuran and nitenpyram; figure 1*a*) excluding thiamethoxam, a precursor of clothianidin [49], on 13 *An. gambiae* nAChRs which were coexpressed by combinations of *An. gambiae α*1 (Ag*α*1, alternatively referred to as Agam*α*1 [50]), Ag*α*2, Ag*α*3, Ag*α*8 and Ag*β*1 subunits with the aid of cofactors AgRIC-3, AgUNC-50 and AgTMX3 in *X. laevis* oocytes.

As these studies uncovered a unique agonist action of dinotefuran on *An. gambiae* nAChRs, we then determined the X-ray crystal structure of this particular compound bound to the AChBP, an established surrogate for the nAChR ligand binding domain (LBD) [51,52]. We report the diverse actions of neonicotinoids on heterologously expressed *An. gambiae* nAChRs, new findings on the mechanism of action of dinotefuran, new insights into the contributions of nAChR subunits and neonicotinoid structural features on vector target-site actions, as well as data on the rate of progress of neonicotinoid knockdown in adult female mosquitoes.

## 2. Results and discussion

### 2.1. Functional expression of *An. gambiae* nAChRs in *X. laevis* oocytes

In *D. melanogaster*, the D*α*1, D*α*2, D*α*3, D*β*1 and D*β*2 nAChR subunits are predominantly expressed in the brain and ventral nerve cord [47]. Of these subunits, D*β*2 shares 83% amino acid sequence identity with the *An. gambiae* Ag*α*8 subunit; a finding similar to the case in honeybees, where Am*α*8 shows 75% identity with D*β*2 [53]. Hence, we tested whether the orthologous *An. gambiae* Ag*α*1, Ag*α*2, Ag*α*3, Ag*α*8 and Ag*β*1 subunits form functional nAChRs in *X. laevis* oocytes with the aid of cofactors AgTMX3, AgRIC-3 and AgUNC-50 (electronic supplementary material, table S1 provides abbreviations for the nAChR subunits and cofactors used in this study and their cDNA accession numbers). These cofactors were deployed as in other insect species (fruit fly, honeybee and bumblebee) where their orthologues have proved to be vital for robust functional nAChR expression [39]. We found that 13 *An. gambiae* nAChR subtypes responded to bath-applied 100 µM ACh (figure 1*d*; electronic supplementary material, S1) and the Ag*β*1 nAChR subunit was vital for robust function, as was the case for fruit fly, honeybee and bumblebee orthologous nAChRs [39], thus confirming a critical role for the *β*1 subunit in forming functional mosquito heteromeric nAChRs (figure 1*d,e*). Also, it should be noted that the Ag*α*1 subunit plays a critical role in enhancing the current amplitude of the response to ACh, indicating the presence of structural features in this subunit contributing to this effect (figure 1*d,e*).

Using nonlinear regression of concentration-response data, we determined ACh affinity for the receptor subtypes by measuring pEC_50_ (= –log EC_50_), where EC_50_ is the concentration (M) giving half the maximal response (electronic supplementary material, table S2). The pEC_50_ values for ACh varied markedly with subunit combinations, with those containing Ag*α*3 and lacking Ag*α*1 and Ag*α*2 subunits exhibiting the highest affinity (figure 1*f*; see Material and methods, and electronic supplementary material, table S3, for statistical analyses using one-way ANOVA).

### 2.2. Diverse neonicotinoid actions on recombinant *An. gambiae* nAChRs

**Figure 2.**
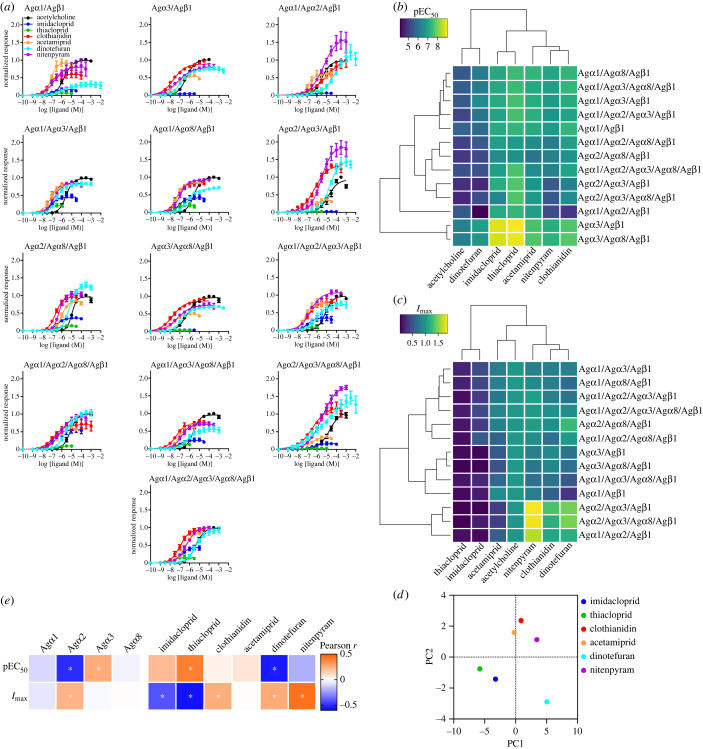
Concentration–agonist activity relationships for ACh and neonicotinoids (imidacloprid, thiacloprid, clothianidin, acetamiprid, dinotefuran and nitenpyram) tested on 13 *An. gambiae* nAChRs expressed in *X. laevis* oocytes and analyses of factors governing agonist activity indices pEC_50_ and *I*_max_. (*a*) Concentration-agonist activity relationships for ACh and neonicotinoids. Each data plot represents the mean ± standard error of the mean (*n* = 5). Curves were fitted by nonlinear regression analysis. (*b,c*) Two dimensional clustering of pEC_50_ (*b*) and *I*_max_ (*c*) values of the neonicotinoids for the 13 *An. gambiae* nAChR subtypes expressed in *X. laevis* oocytes. Imidacloprid and thiacloprid containing the E-bridge were paired, while acetamiprid, clothianidin and nitenpyram form a separate group. Dinotefuran, showing unique binding features, forms an outgroup with ACh. Thus, the E-bridge contributes to enhancing the affinity of neonicotinoids. For subunit combinations, neonicotinoids exhibited the highest affinity for the Ag*α*3/Ag*β*1 and Ag*α*3/Ag*α*8/Ag*β*1 nAChRs with no Ag*α*2 subunit, indicating that the Ag*α*2 subunit has an affinity reducing effect. (*d*) Principal component scores for the neonicotinoids. Combined analyses of pEC_50_ and *I*_max_ pointed to unique features of dinotefuran which was plotted alone in the second quadrant. (*e*) Correlation of the agonist potency indices with the nAChR subunits and the neonicotinoids. The blueish colour in pEC_50_ and reddish colour in *I*_max_ of the Ag*α*2 subunit indicated that neonicotinoids have a lower affinity for those subtypes which include Ag*α*2, while increasing the efficacy. The Ag*α*3 subunit increases the affinity while it has no clear effect on efficacy. For *I*_max_ of compounds, imidacloprid and thiacloprid generally showed lower efficacy than clothianidin, dinotefuran and nitenpyram.

Next, we evaluated the affinity (pEC_50_) and efficacy (*I*_max_) of 6 neonicotinoids (imidacloprid, thiacloprid, clothianidin, acetamiprid, dinotefuran and nitenpyram; figure 1*a*) for the 13 ACh-responsive *An. gambiae* nAChRs subtypes (figure 2*a*; electronic supplementary material, S2–S7 for nAChR responses to neonicotinoids; electronic supplementary material, table S2 for agonist activity indices; electronic supplementary material, table S4 for statistical analyses). Based on pEC_50_ values, thiacloprid and imidacloprid tended to show higher affinity than the others for each nAChR (electronic supplementary material, table S2), while acetamiprid and clothianidin exhibited moderate affinity. By contrast, based on *I*_max_ values, acetamiprid, clothianidin, dinotefuran and nitenpyram tended to show higher efficacy than imidacloprid and thiacloprid for nAChRs. We also noted that dinotefuran and nitenpyram were super agonists (defined by an *I*_max_ greater than 1, i.e. the peak current amplitude of the nAChR response to these two neonicotinoids is greater than that of ACh observed at saturating concentrations) in the case of Ag*α*2-containing nAChR subtypes (figure 2*a*; electronic supplementary material, table S2). *I*_max_ may reflect the efficacy of several nAChRs. However, the Ag*α*1/Ag*α*2/Ag*β*1, Ag*α*1/Ag*α*3/Ag*β*1 and Ag*α*1/Ag*α*8/Ag*β*1 nAChRs largely represent their own features in terms of the interactions with neonicotinoids distinct from the feature of Ag*α*1/Ag*β*1 nAChR (see following results) since the contributions of the Ag*α*2/Ag*β*1, Ag*α*3/Ag*β*1 and Ag*α*8/Ag*β*1 nAChRs to the total ligand induced-nAChR responses are very small or even zero (figure 1*e*). For all the nAChR subunit combinations, the difference in expression levels would be cancelled by normalization to the ACh-induced response amplitude. Whatever the factors underlying *I*_max_, the notion holds that the neonicotinoid structural features enhancing the affinity have opposite effects on efficacy.

### 2.3. Clustering and multivariate analyses of *An. gambiae* nAChR subtypes and neonicotinoid features

To understand nAChR subunit and ligand factors underpinning agonist activity, we first analysed the pEC_50_ and *I*_max_ values of neonicotinoids by hierarchical two dimensional (2D) clustering when each subunit or ligand was characterized by an indicator variable which takes 1 and 0 for the presence and absence of each factor, respectively [47] (figure 2*b,c*). For the pEC_50_ values, neonicotinoids clustered into an ethylene bridge (E-bridge: -CH_2_-CH_2_- linkage)-containing group (imidacloprid and thiacloprid), and those without an E-bridge (acetamiprid, clothianidin and nitenpyram). However, dinotefuran was quite distinct from the other groups with respect to nAChR actions in that it showed the lowest affinity but high efficacy (figure 2*b*). Imidacloprid and thiacloprid, containing the E-bridge, tended to show higher agonist affinity than those compounds lacking the bridge (figure 2*b*), probably reflecting the reliance on CH-π interactions of the E-bridge hydrogens with the tryptophan in loop B.

The hierarchical 2D clustering of *I*_max_ separated the nAChR subtypes into high and low efficacy groups (figure 2*c*). Most neonicotinoids exhibited high efficacy for the nAChRs containing the Ag*α*2 subunit. Imidacloprid and thiacloprid, both possessing the E-bridge, showed a lower efficacy than those without the bridge, probably due to the CH-π interactions preventing the neonicotinoids from flexible binding to the orthosteric site, which twists in response to agonist binding [54]. Clothianidin and nitenpyram both acted as super agonists on the Ag*α*2/Ag*α*3/Ag*β*1 and Ag*α*2/Ag*α*3/Ag*α*8/Ag*β*1 nAChR subtypes (figure 2*c*; electronic supplementary material, table S2), supporting the grouping of the no E-bridge neonicotinoids (figure 2*c*) observed in affinity-based clusters, providing further support for the diversity of action of neonicotinoids on *An. gambiae* nAChRs.

Next, we performed principal component analyses (PCAs) for the pEC_50_ and *I*_max_ data sets to examine the similarity/diversity of *An. gambiae* nAChR and compound features revealed by the 2D clustering. The Ag*α*1/Ag*β*1, Ag*α*1/Ag*α*3/Ag*β*1 and Ag*α*1/Ag*α*8/Ag*β*1 nAChR subtypes are grouped together in the pEC_50_ data set but separated in the case of the *I*_max_ data set (electronic supplementary material, figure S8*a*, tables S2 and S4). Also, the Ag*α*2/Ag*α*8/Ag*β*1 and the Ag*α*1/Ag*α*2/Ag*α*8/Ag*β*1 nAChRs are similar in terms of pEC_50_ profiles but separated in the context of *I*_max_ profiles (electronic supplementary material, figure S8*a*), indicating that the *An. gambiae* nAChRs studied have their own distinctive features, including diverse pharmacological responses to the 6 neonicotinoids.

For the neonicotinoid features, PCA of the pEC_50_ and *I*_max_ data sets distinguished between compounds with an E-bridge and those without it and also placed dinotefuran in a separate category, supporting the characterization shown by the 2D clustering (figure 2*d* for all the agonist activity data set (pEC_50_ + *I*_max_); electronic supplementary material, figure S8*b* for each pEC_50_ and *I*_max_ set).

Finally, to test for linear correlation between data on *An. gambiae* nAChR subunits and neonicotinoid features influencing the agonist activity indices, we calculated the Pearson coefficients of pEC_50_ and *I*_max_ (figure 2*e*). This analysis showed that the presence of Ag*α*2 reduced affinity whereas the presence of Ag*α*3 enhanced affinity (figure 2*e*; electronic supplementary material, table S5). Also, thiacloprid structure increased affinity, whereas dinotefuran structure lowered it (figure 2*e*; electronic supplementary material, table S5). For *I*_max_, the Ag*α*2 subunit increased the efficacy, while the other subunits had no significant contribution to the index. Imidacloprid and thiacloprid showed negative correlations with *I*_max_, whereas clothianidin, dinotefuran and nitenpyram had positive effects on the values in Pearson correlation analyses (figure 2*e*; electronic supplementary material, table S5). As such, the E-bridge neonicotinoids, imidacloprid and thiacloprid, contrast with the non-E-bridge neonicotinoids, dinotefuran and nitenpyram, in the correlations with affinity and efficacy (see red and blue colours for positive and negative correlations, respectively). Notably, clothianidin has a significant positive effect on efficacy with no significant negative effect on efficacy for the nAChRs tested (figure 2*e*), supporting its selection for managing *An. gambiae*.

### 2.4. Crystal structure of the AChBP-dinotefuran complex

**Figure 3.**
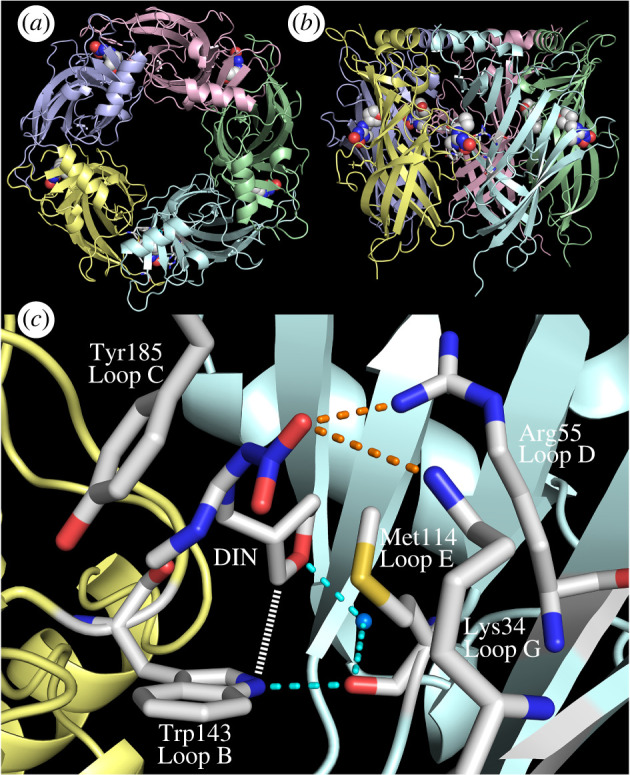
Crystal structure of the Q55R mutant of *Ls*-AChBP in complex with dinotefuran. The mutation was made to mimic insect nAChR basic residues located in loop D of the *β*1 subunits [15,25,27,28,30,32,34,71]. (*a*) Top and (*b*) side views of the crystal structure showing that *Ls-*AChBP assembles to form a homo-pentamer and that dinotefuran bound to all the five orthosteric sites. (*c*) Expanded view of the interactions of dinotefuran with key amino acids at the binding site. Main chains of principal and complementary proteins are coloured pale yellow and pale cyan, respectively. Dinotefuran and the key amino acids are represented as sticks, and carbons, nitrogens, oxygens and sulfur are coloured white/grey, blue, red and yellow, respectively. A water molecule involved in the hydrogen bond networks is represented as a sphere and coloured marine blue. Hydrogen bonds and electrostatic interactions represented as dotted lines are coloured cyan and orange, respectively. The CH-N interactions are represented as a white dashed line. The X-ray crystal structure revealed that the nitro group interacted with Lys34 in loop G and Arg55 in loop D of the complementary subunit, while its guanidine group stacked with Tyr185 in loop C. Uniquely, the tetrahydrofuran ring interacts with nitrogen of Trp143 loop C by CH-N interactions which are not seen in the AChBP complexed with imidacloprid, clothianidin, thiacloprid and the nitromethylene analogue of imidacloprid [32].

In an attempt to clarify the divergent mechanism of dinotefuran's interactions with the *An. gambiae* nAChRs tested, we cocrystallized it with the *Lymnaea stagnalis* AChBP (*Ls-*AChBP), which is not a nAChR but has been widely used as a surrogate for LBD in nAChR interactions [51,55,56], since no insect nAChR has been crystallized to date. In this experiment, we employed the *Ls-*AChBP Q55R mutant as it mimics insect nAChRs in possessing the basic residue in loop D [32]. Dinotefuran bound to all five orthosteric sites of the protein as observed for imidacloprid, thiacloprid, clothianidin and the nitromethylene analogue of imidacloprid [32,57] (figure 3*a,b*; electronic supplementary material, table S6). Of the dinotefuran stereoisomers, only the *S-*isomer cocrystallized with the *Ls*-AChBP (figure 3*c*), in line with the finding that the *S-*isomer was more potent than the *R*-isomer in binding to the housefly (*Musca domestica* nAChRs) [58]. In the Q55R mutant of *Ls-*AChBP, the guanidine moiety of dinotefuran stacked with Tyr185 in loop C (figure 3*c*) as in other neonicotinoids [32,57]. The nitro group interacted electrostatically with the Arg55 in loop D and Lys34 in loop G (figure 3*c*), confirming that these basic residues in loop D and loop G generally play an important role in the selective insect nAChR−neonicotinoid interactions [15]. The non-aromatic tetrahydrofuran ring is a unique structure of dinotefuran not seen in other neonicotinoids (figure 1*a*). The tetrahydrofuran ring oxygen formed a hydrogen bond via water with the indole ring NH of Trp143 in loop B and the main chain carbonyl of Met114 in loop E (figure 3*c*) as in the cases of the pyridine/thiazole nitrogen in the other neonicotinoids [32,57]. Nevertheless, the tetrahydrofuran ring hydrogens undergo CH-N electrostatic interactions, which are not seen in the crystal structures of the Q55R mutant of *Ls*-AChBP in complex with imidacloprid, thiacloprid and clothianidin [32]. Also, the guanidine NH of dinotefuran did not form a hydrogen bond with the main chain carbonyl of Trp143 (figure 3*c*), as was the case for clothianidin [32]. As such, the structural information supports the unique experimental binding interactions of dinotefuran with the *An. gambiae* nAChRs, with the caveat that the AChBP is only a surrogate, albeit useful model of the nAChR LBD [32,55,57,59].

### 2.5. Relationship of the target site actions with the knockdown rate of neonicotinoids

**Figure 4.**
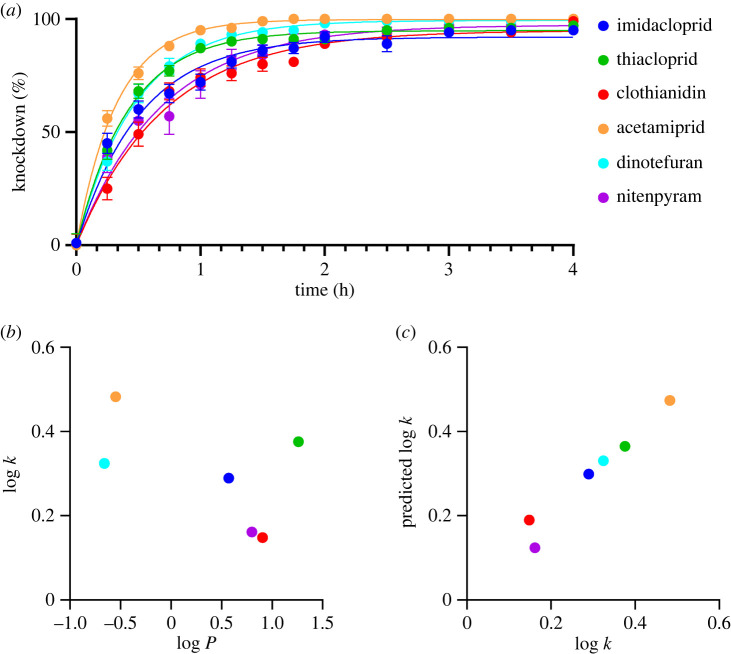
Progress of knockdown of neonicotinoids for adult females of *An. gambiae* mosquitoes (*An. gambiae* s.l. (N'gousso strain *An. coluzzi*)) and the features of neonicotinoids. (*a*) Time-dependent development of knockdown following treatment with the neonicotinoids. (*b*) Relationship of log *k* (*k* is rate of progress of knockdown symptom) and log *P* (*P* is 1-octanol/water partition coefficient). (*c*) Correlation of the predicted and measured log *k* values. The high correlation of the predicted and measured values suggests a prominent role for the Ag*α*1/Ag*α*2/Ag*α*8/Ag*β*1 nAChR in determining the rate of progress of the knockdown symptom in adult females of *An. gambiae*.

Finally, we investigated the factors governing variations in the rate of insecticide knockdown in adult female mosquitoes when exposed to fixed doses of each neonicotinoid. We determined a knockdown rate constant *k* from the time-dependent progress of mosquito knockdown by fitting the data to a single exponential curve (see Material and methods for detail; see figure 4*a* and electronic supplementary material, table S7 for data). We then examined correlation of log *k* with log *P* (*P* is 1-octanol/water partition coefficient, electronic supplementary material, table S7) representing hydrophobicity of the neonicotinoids. We pursued this approach because the knockdown rate of pyrethroids is well known to be relatable to compound hydrophobicity, which affects both penetration and transport of compounds from the contact site to the target protein [60]. For the neonicotinoids studied, the log *k* value appeared to have a negative correlation with log *P* (figure 4*b*), but the correlation was not significant, suggesting the involvement of other factors in determining the knockdown rate (figure 4*b*). We therefore analysed variations of log *k* with pEC_50_ or *I*_max_ values and log *P* by multiple linear regression, resulting in equation (2.1) as the best one with the highest adjusted correlation coefficient *r* and the smallest Akaike's information criterion [61] with a correction for small sample sizes (AICc) [62] which estimates prediction error (the lower the AICc, the better the model) as follows.

2.1 $$\begin{array}{rl} \log\;k= & -0.170(95\text{\%}\;\mathrm{CI}-0.247--0.0916)\log\;P\\ & -0.383(95\text{\%}\mathrm{CI}-0.573--0.193)I_{\max}\mathrm{Ag}\alpha 1/\mathrm{Ag}\alpha 2/\mathrm{Ag}\alpha 8/\mathrm{Ag}\beta 1\mathrm{nAChR}\\ & +0.571(95\text{\%}\mathrm{CI}\;0.462\text{–}0.763), \end{array}$$

adjusted *r*^2^ = 0.918, *F*_2, 3_ = 28.9 and AICc = 4.18 (figure 4*c*; electronic supplementary material, table S8). Running equation (2.1) indicated that the lower the hydrophobicity and the lower the efficacy for the Ag*α*1/Ag*α*2/Ag*α*8/Ag*β*1 nAChR, the faster the neonicotinoids knock down the mosquitoes. Neither pEC_50_ nor *I*_max_ values for the other *An. gambiae* nAChR subtypes resulted in significant regression with log *k* even if the log *P* term was added (*p* > 0.05, electronic supplementary material, table S8), suggesting that the Ag*α*1/Ag*α*2/Ag*α*8/Ag*β*1 nAChR subtype, though its presence in native neurons controlling the mosquito flight locomotion awaits evidence, plays a prominent role in the mosquito neurobiology and hence suppression of and accessibility to this nAChR subtype determines the knockdown rate. More studies are needed to explore the detailed functional roles of the Ag*α*1/Ag*α*2/Ag*α*8/Ag*β*1 nAChR in the *An. gambiae* disease vector, but it is our working hypothesis that hydrophobicity as well as antagonist actions of neonicotinoids are key to their ability to knockdown adult female *Anopheles* mosquitoes.

In conclusion, we have obtained robust, heterologous, functional expression of 13 different *An. gambiae* nAChR in *X. laevis* oocytes and clarified nAChR subunit contributions and compound properties of 6 neonicotinoids underpinning the affinity and efficacy of this class of nAChR-targeting compounds including one pre-approved by WHO for mosquito control. We found that the Ag*α*3 subunit enhanced neonicotinoid affinity, whereas the Ag*α*2 subunit reduced it. We showed previously that reducing the *α*2 subunit gene expression led to enhanced neonicotinoid sensitivity in adult *D. melanogaster* [47]*.* Thus, we hypothesize that either reducing Ag*α*3 gene expression, or increasing Ag*α*2 gene expression, or both, can lead to neonicotinoid resistance. Dinotefuran interacted directly with the mosquito nAChR likely through hydrogen bond formation and CH-N interactions of the tetrahydrofuran ring, exhibiting a unique type of agonist action. Quantitative analyses pointed to compound hydrophobicity and antagonist actions of neonicotinoids on an *An. gambiae* nAChR subtype governing the rate of knockdown. These findings aid our understanding of the target-site actions of neonicotinoids including clothianidin and dinotefuran, both of which may have a role to play in the control of the *An. gambiae* malaria vector.

## 3. Material and methods

### 3.1. Chemicals

ACh chloride and atropine sulfate were purchased from MilliporeSigma (USA). All the neonicotinoids (Imidacloprid, thiacloprid, clothianidin, dinotefuran, nitenpyram and acetamiprid) were purchased from FujiFilm Wako Pure Chemical (Japan). These reagents were used without further purification.

### 3.2. cRNA preparation

cRNA was prepared from the pcDNA3.1 (+) plasmid vector (Thermo Fisher Scientific, USA) containing each nAChR subunit or cofactor cDNA using mMESSAGE mMACHINE T7 ULTRA Transcription Kit (Thermo Fisher Scientific).

### 3.3. cRNA injection into *X. laevis* oocytes

The oocytes were treated with collagenase (Type IA, MilliporeSigma) in Ca^2+^-free standard oocyte saline (Ca^2+^-free SOS) containing 100 mM NaCl, 2 mM KCl, 1 mM MgCl_2_, 5 mM 4-(2-hydroxyethyl)-1-piperazineethanesulfonic acid (HEPES), pH 7.6 for 40 min at room temperature. Isolated oocytes were then transferred to SOS containing 100 mM NaCl, 2 mM KCl, 1.8 mM CaCl_2_, 1 mM MgCl_2_, 5 mM HEPES, pH 7.6. The follicle layers removed manually using forceps. 50 nl of cRNA mixtures encoding various *An. gambiae* nAChR subunits, always together with three cofactors (AgRIC-3, AgUNC-50 and AgTMX3), were injected into oocytes at concentrations 0.1 ng nl^−1^. Finally, oocytes were incubated in SOS supplemented with 2.5 mM sodium pyruvate, 100 units ml^−1^ penicillin, 100 µg ml^−1^ streptomycin, 20 µg ml^−1^ gentamycin and 4% horse serum (heat inactivated, Thermo Fisher Scientific) at 16°C for 2–5 days prior to commencing electrophysiology experiments.

### 3.4. Electrophysiology

Two-electrode, voltage-clamp electrophysiology was used to investigate *An. gambiae* nAChRs expressed in *X. laevis* oocytes. Oocytes were placed in a Perspex recording chamber and voltage clamped at a membrane potential of −100 mV and perfused with SOS containing 0.5 µM atropine at a flow rate of 7–10 ml min^−1^ [39,63]. Responses to ACh and neonicotinoids were recorded as inward currents and analysed offline using pCLAMP software (Molecular Devices, USA). Recordings were repeated at each compound dose (*n* = 5 using oocytes from at least two female frogs). When comparing the peak current amplitude of the ACh-induced response, we measured the response amplitude of 10 oocytes (5 oocytes from each of two different female frogs).

### 3.5. Analysis of electrophysiological data

Peak current amplitude of the response to ACh and neonicotinoids versus concentration were measured and fitted by nonlinear regression analysis using Prism software (GraphPad Software, USA) according to the following equation.

3.1 $$Y=\frac{I_{\max}}{1+10^{({\mathrm{logEC}}_{50}-\mathrm{logX})n_{H}}}.$$

In this equation, Y is peak current amplitude of the ACh or neonicotinoid response normalized to the maximum peak amplitude of the ACh-induced response, where X is ligand concentration (M), *n*_H_ is the Hill coefficient and *I*_max_ is normalized ACh maximum response.

### 3.6. Multivariate analyses

Multivariate analyses of pEC_50_ and *I*_max_ values were performed using Prism software using indicator variables for subunits and neonicotinoids. For example, to represent the Ag*α*1/Ag*α*2/Ag*β*1 nAChR, we set indicator variables 1, 1, 0, 0, and 1 for the Ag*α*1, Ag*α*2, Ag*α*3, Ag*α*8, and Ag*β*1 subunits, respectively. Similarly, we assign 1 to represent a test of compounds as performed previously [47]. 2D-Hierachial clustering was performed using the *R* package's gplots and RColorBrewer with Viridis color gradient. Pearson correlation coefficients of the pEC_50_ and *I*_max_ values were calculated by Prism software.

### 3.7. Preparation of *Ls-*AChBP and cocrystallization with dinotefuran

The Q55R mutant of the *Ls-*AChBP was over-expressed in *Pichia pastoris*, deglycosylated and purified, as described previously [32]. The protein was cocrystallized with 1 mM dinotefuran in precipitant solution composed of 16.5–18.0% PEG4000 and 0.2 M sodium citrate (pH 5.4) at 20°C. X-ray diffraction data were obtained at SPring-8 BL26B1 beamline at 100 K using a CCD detector RAYONIX MX225HE. The diffraction dataset was first processed using XDS [64] and Aimless (CCP4: supported program) [65], and the initial phase was determined by molecular replacement using MOLREP [66] with a protein coordinate of 2ZJU. Refinement of the structure model was performed using REFMAC5 [67], and manual model building was performed with Coot [68] (electronic supplementary material, table S6).

### 3.8. Bioassays on female *An. gambiae*

Neonicotinoid bioassays were carried out following the guidelines from the Centers for Disease Control and Prevention [69]. Briefly, neonicotinoids were dissolved and diluted to a fixed concentration of 4 µg ml^−1^ in acetone containing 0.11% methylated rapeseed oil (RME). 250 ml Wheaton bottles were then coated with an even distribution of 1 ml insecticide, through inversion and then rolling until acetone had evaporated, and then left overnight in a horizontal position. Up to 25 female 3–5-day-old *An. gambiae* s.l. (N'gousso strain *An. coluzzi*) mosquitoes were added to each bottle, the bottle sealed with cap, and the rate of knockdown measured over 4 h. Knockdown was counted as mosquitoes unable to stand or fly when bottle was gently agitated. Counts were taken every 15 min for the first 2 h, and then every 30 min. Each insecticide was assayed in duplicate, and the experiment was repeated (*n* = 5). For each repeat, mosquitoes were exposed to acetone (0.11% RME) treated bottles as controls.

The knockdown (KD) rate constant *k* (h^−1^) was determined according to the following equation.

3.2 $$\mathrm{KD}\;(t)={\mathrm{KD}}_{\mathrm{plateau}}(1-e^{-kt}).$$

In equation (3.2), KD (*t*) and KD_plateau_ are knocked down mosquito percentages at time *t* (h) and plateau, respectively.

### 3.9. Data analysis

Differences of agonist activities on the nAChRs (pEC_50_, *I*_max_) were analysed by one-way ANOVA at a level of false discovery rate (FDR) [70] *q* < 0.05. Pearson correlation coefficients were analysed by 95% confidence interval (95% CI, two-tailed), while the multiple regression and correlation coefficients of each parameter were analysed by *F*-values and 95% CI (two-tailed), respectively.

## Data Availability

The accession numbers of the *A. gambiae* nAChR subunits and cofactors that aid robust functional expression are as follows: (subunits) Ag*α*1 (XM_311918), Ag*α*2 (XM_311921), Ag*α*3 (XM_310786), Ag*α*8 (XM_311925), Ag*β*1 (XM_309158); (cofactors) AgRIC-3 (XM_313931), AgUNC-50 (XM_312002) and AgTMX3 (XM_315438) (electronic supplementary material, table S1). The current amplitude of the responses to ACh of the nAChRs tested are available from electronic supplementary material (figure 1*e* current amplitude data.csv). All data used for multivariate analyses are available from electronic supplementary material [72].
