# Correction to: ‘Unravelling nicotinic receptor and ligand features underlying neonicotinoid knockdown actions on the malaria vector mosquito *Anopheles gambiae*’ (2024), by Ito *et al.*

**DOI:** 10.1098/rsob.250033

**Published:** 2025-04-10

**Authors:** Ryo Ito, Masaki Kamiya, Koichi Takayama, Sumito Mori, Rei Matsumoto, Mayuka Takebayashi, Hisanori Ojima, Shota Fujimura, Haruki Yamamoto, Masayuki Ohno, Makoto Ihara, Toshihide Okajima, Atsuko Yamashita, Fraser Colman, Gareth J. Lycett, David B. Sattelle, Kazuhiko Matsuda

**Affiliations:** ^1^ Graduate School of Agriculture, Kindai University, Nara, 631-8505, Japan; ^2^ Department of Applied Biological Chemistry, Faculty of Agriculture, Kindai University, Nara, 631-8505, Japan; ^3^ Institute of Scientific and Industrial Research, Osaka University, Suita, Osaka 567-0047, Japan; ^4^ Graduate School of Medicine, Dentistry and Pharmaceutical Sciences, Okayama University, Okayama, 700-8530, Japan; ^5^ Liverpool School of Tropical Medicine, Liverpool L3 5QA, UK; ^6^ University College London, London WC1E 6JF, UK; ^7^ Agricultural Technology and Innovation Research Institute, Kindai University, Nara, 631-8505, Japan

**Keywords:** malaria, *Anopheles gambiae*, neonicotinoids, nicotinic acetylcholine receptors, knockdown

**Figure:**
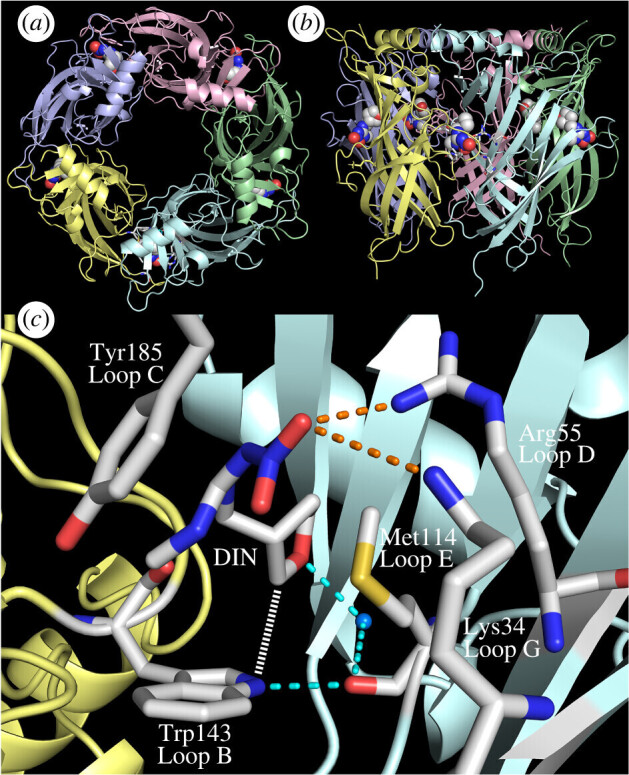

Although the biological data we presented in the original paper are correct, there is an error in the calculated log *P* values for certain compounds, now corrected in this Corrigendum. One conclusion in the original paper as published, which states that log *P* and Imax are negatively correlated with log *k*, is incorrect and we clarify that in this Corrigendum. The corrected calculations still indicate that the Agα1/Agα2/Agα8/Agβ1 nAChR is the most important factor determining the rate constant, as described in the original paper. This has been corrected in the text below.

## 2. Results and discussion

### 2.1 Relationship of the target site actions with the knockdown rate of neonicotinoids

Finally, we investigated the factors governing variations in the rate of insecticide knockdown in adult female mosquitoes when exposed to fixed doses of each neonicotinoid. We determined a knockdown rate constant *k* from the time-dependent progress of mosquito knockdown by fitting the data to a single exponential curve (see ‘Material and Methods’ for detail, figure 4A and electronic supplementary material, table S7 for data). We then examined the correlation of log *k* with log *P* (*P* is 1-octanol/water partition coefficient, electronic supplementary material, table S7) representing hydrophobicity of the neonicotinoids. We pursued this approach because the knockdown rate of pyrethroids is well known to be relatable to compound hydrophobicity, which affects both penetration and transport of compounds from the contact site to the target protein (60). For the neonicotinoids studied, the log *k* value appeared to have no correlation with log *P* (figure 4B), suggesting the involvement of other factors in determining the knockdown rate (figure 4B). We, therefore, analysed variations of log *k* with pEC_50_ or *I*_max_ values and log *P* by partial least squares (PLS) and found that the pEC_50_ values (figure 4C) better accounted for the log *k* values compared with *I*_max_ values (figure 4D). We further examined which nAChR subtypes contributed to the rate of development of *Anopheles gambiae* knockdown symptoms (figure 4E,F). The pEC_50_ values for the Agα1/Agα2/Agα8/Agβ1 nAChR subtype, followed by those of Agα1/Agβ1, Agα1/Agα2/Agβ1, Agα1/Agα3/Agα8/Agβ1 and Agα1/Agα2/Agα3/Agα8/Agβ1 nAChR subtypes indicated that affinity for these nAChR subtypes determines the *An. gambiae* knockdown rate (figure 4E). Although more studies are needed to explore the detailed functional roles of the Agα1/Agα2/Agα8/Agβ1 nAChR in the *An. gambiae* disease vector, it is our working hypothesis that agonist actions of neonicotinoids on this nAChR subtype are key to their ability to knockdown adult female *An. gambiae* mosquitoes.

**Figure 4.**
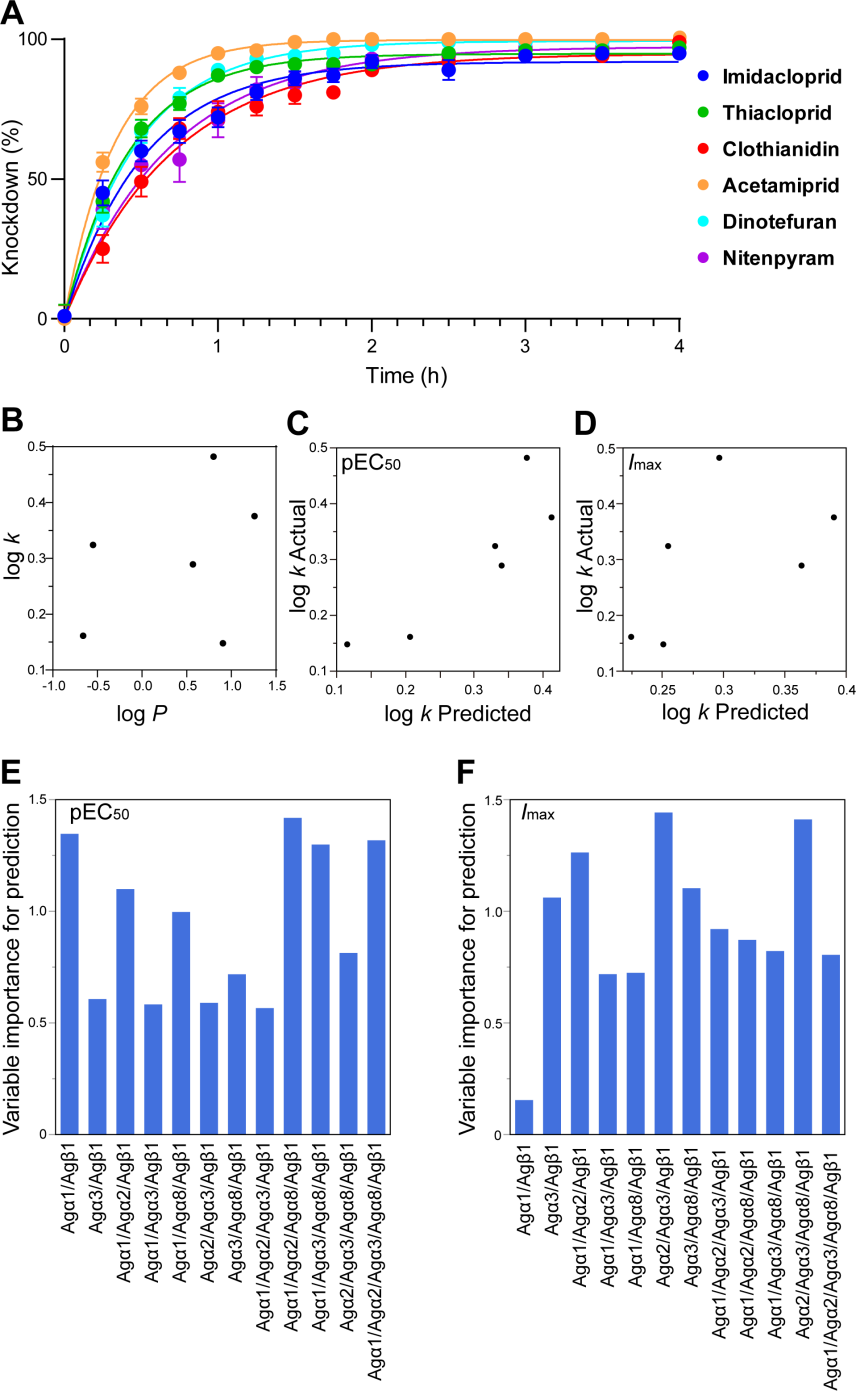
Progress of knockdown of neonicotinoids for adult females of *Anopheles gambiae* mosquitoes (*An. gambiae* s.l. (N’gousso strain *Anopheles coluzzi*)) and the features and target site actions of neonicotinoids. (A) Time-dependent development of knockdown following treatment with the neonicotinoids. (B) Relationship of log *k* (*k* is rate constant of progress of knockdown symptom) and log *P* (*P* is 1-octanol/water partition coefficient). (C) Correlation of the predicted by pEC_50_ values and measured log *k* values. (D) Correlation of the predicted by *I*_max_ values and measured log *k* values. Higher correlation of predicted and measured values was observed for pEC_50_ (C) than for *I*_max_ (D). (E) The importance of pEC_50_ values for each nAChR subtype in determining the log *k* values. (F) The importance of *I*_max_ values for each nAChR subtype in determining the log *k* values. The Agα2/Agα8/Agβ1 nAChR was omitted from (E,F) because its response amplitude to the neonicotinoids tested was very small. The partial least squares analysis results shown in (E,F) indicate a prominent role for the Agα1/Agα2/Agα8/Agβ1 nAChR in determining the rate of progress of knockdown in adult females of *An. gambiae*.

In conclusion, we have obtained robust, heterologous, functional expression of 13 different *An. gambiae* nAChRs in *Xenopus laevis* oocytes and clarified nAChR subunit contributions and compound properties of six neonicotinoids underpinning the affinity and efficacy of this class of nAChR-targeting compounds, including one pre-approved by the World Health Organization for mosquito control. We found that the Agα3 subunit enhanced neonicotinoid affinity, whereas the Agα2 subunits reduced it. We showed previously that reducing the α2 subunit gene expression led to enhanced neonicotinoid sensitivity in adult *Drosophila melanogaster* (47). Thus, we hypothesize that either reducing Agα3 gene expression or increasing Agα2 gene expression, or both, can lead to neonicotinoid resistance. Dinotefuran interacted directly with the mosquito nAChR likely through hydrogen bond formation and CH–N interactions of the tetrahydrofuran ring, exhibiting a unique type of agonist action. Quantitative analyses pointed to compound agonist actions of neonicotinoids on *An. gambiae* nAChR subtype governing the rate of knockdown. These findings aid our understanding of the target-site actions of neonicotinoids including clothianidin and dinotefuran, both of which may have a role to play in the control of the *An. gambiae* malaria vector.

## 3. Methods

### 3.1. Data analysis

Differences in agonist activities on the nAChRs (pEC_50_, *I*_max_) were analysed by one-way analysis of variance at a level of false discovery rate (70) *q* < 0.05. Pearson correlation coefficients were analysed by 95% confidence intervals (95% CI, two-tailed), while the multiple regression and correlation coefficients of each parameter were analysed by *F* values and 95% CI (two-tailed), respectively. PLS analyses were conducted using the JMP software (USA).

